# Analysis of Antioxidant Activity and Volatile Components in Rapeseed Flower-Enriched Persimmon Wine

**DOI:** 10.3390/foods14101804

**Published:** 2025-05-19

**Authors:** Zhijie Li, Kaishuo Sun, Yanyan Wang, Fang Yu, Zhiwen Liu

**Affiliations:** 1 School of Biological Engineering, Dalian Polytechnic University, Dalian 116034, China; 220210710000165@xy.dlpu.edu.cn (Z.L.); sks18236691936@outlook.com (K.S.); wang_yy@dlpu.edu.cn (Y.W.); 2 College of Bioscience and Biotechnology, Shenyang Agricultural University, Shenyang 110866, China; yufang@dlpu.edu.cn

**Keywords:** persimmon wine, rapeseed flower, antioxidant, flavoring substance, molecular dynamics simulation

## Abstract

The quality of persimmon wine is closely related to various compounds, including polysaccharides. Polysaccharides are an essential class of macromolecules that modulate the wine’s chemical and physical characteristics by influencing the colloidal state or interacting with other compounds through non-covalent bonds. Polyphenols, on the other hand, exhibit antioxidant properties and effectively neutralize free radicals. This study employed Luotian sweet persimmons and Brassica napus (rapeseed) as core ingredients for producing functional fermented wine. Using GC-MS, rapeseed polysaccharides were subjected to trifluoroacetic acid hydrolysis and then derivatized via silylation for qualitative analysis of their monosaccharide composition. Molecular docking and molecular dynamics simulations were performed to provide molecular-level insights into the interactions between D-glucopyranose from rapeseed polysaccharides and quercetin, a polyphenol present in persimmon wine. The objective was to explore the binding mechanisms of these compounds during fermentation and to assess how these molecular interactions in-fluence the wine’s flavor and stability. In addition, volatile flavor compounds in two types of persimmon wine (pure persimmon wine and oleoresin-enriched persimmon wine) were qualitatively and quantitatively analyzed using headspace solid-phase microextraction (SPME) combined with gas chromatography–mass spectrometry (GC-MS). The results reveal that D-glucopyranose forms hydrogen bonds with quercetin, modulating its redox behavior and thereby enhancing the antioxidant capacity of persimmon wine. The results from four in vitro antioxidant assays, including DPPH, ABTS, FRAP, and vitamin C analysis, demonstrate that the addition of rapeseed flowers improved the antioxidant activity of persimmon wine. HS-SPME-GC-MS analysis revealed that esters, alcohols, and aldehydes were the primary components contributing to the aroma of persimmon wine. Persimmon wines with varying levels of oleoresin addition exhibited significant differences in the contents of key compounds, which subsequently influenced the aroma complexity and flavor balance. In conclusion, these findings provide reliable data and a theoretical foundation for understanding the role of rapeseed flower in regulating the aroma profile of persimmon wine. These findings also offer theoretical support for a deeper understanding of the fermentation mechanisms of persimmon wine while providing practical guidance to optimize production processes, ultimately improving both product flavor and stability. This study fills a critical academic gap in understanding microscopic molecular interactions during fermentation and offers a novel perspective for innovation in the fermented food industry.

## 1. Introduction

*Brassica napus* L. (rapeseed), a key oilseed crop in China, is extensively cultivated nationwide and serves as a critical resource for food processing and industrial applications [1]. Rapeseed is predominantly utilized as edible oil and vegetable products, while its biochemical profile includes vitamin E, polypeptides, polysaccharides, and bioactive compounds. Vitamin E, an essential micronutrient for humans and animals, cannot be synthesized endogenously and requires dietary intake [2]. Recent studies have highlighted rapeseed flowers and persimmon wine as promising candidates for bioactive compound extraction due to their phytochemical richness. Rapeseed flowers and pollen contain diverse bioactive components with high nutritional value, alongside their ornamental significance [3]. Rapeseed pollen, characterized by its distinct yellow pigmentation, is rich in amino acids, proteins, fatty acids, vitamins, polyphenols, flavonoids, and bioactive trace elements. Rapeseed flowers demonstrate exceptional nutraceutical potential, with documented therapeutic effects against arteriosclerosis, prostate disorders, cardiovascular diseases, hepatic dysfunction, anemia, and diabetes [4]. Rapeseed flowers are a source of polysaccharides, flavonoids, and bioactive compounds, including eight essential amino acids and growth-critical macro-/microelements, positioning them as ideal substrates for functional food development [5]. In recent years, the application value of rapeseed has expanded significantly, and the in-depth exploration of its natural active ingredients has brought widespread attention to its comprehensive utilization. Using canola flowers, sprouts, and pollen as raw materials, Chinese research teams have developed a variety of products, including canola tea, sprouts, skincare products, functional biscuits, and cultural and creative items with health benefits. These developments not only enhance the economic value of canola but also support rural revitalization through tourism commercialization and potted flower cultivation.

In contrast, persimmons are recognized as functional foods owing to their high nutrient density, including organic acids, minerals, and bioactive metabolites [6]. Persimmons are processed into value-added products (e.g., dried persimmons, beverages, vinegar, persimmon energy bars, and wine), though current research prioritizes optimizing dehydration techniques for dried persimmon production [7,8]. Persimmon wine, a traditional fruit beverage, is favored for its distinct flavor profile and nutrient-rich composition [9]. It is enriched with polyphenolic compounds, like ellagic acid and quercetin, the latter of which exhibits potent antioxidant, anti-inflammatory, and anti-allergic properties. Plant-derived phenolic compounds, particularly prevalent in fruit wines, are recognized for their antioxidant and antimicrobial efficacy, attributable to polyphenol-rich profiles [10]. Polyphenols, like catechin and epicatechin, demonstrate antidiabetic potential, whereas flavonoids, such as myricetin, quercetin, and kaempferol, show antihypertensive effects [11]. Quercetin, a dominant polyphenol in fruit wines, modulates persimmon wine’s sensory and qualitative attributes through monosaccharide interactions [6]. These interactions may reshape the final product’s properties by altering fermentation dynamics, ethanol yield, and flavor component ratios. Critically, such interactions may inhibit wine oxidation and stabilize quercetin, synergistically enhancing the wine’s antioxidant capacity. The publication describes the link between polyphenolic substances in persimmon wine and the antioxidant activity of polysaccharides derived from rapeseed flower. The primary methods commonly used to assess antioxidant activity in vitro include the DPPH, ABTS, and FRAP assays [12]. Polyphenols in persimmon wine exhibit significant antioxidant activity, with higher concentrations corresponding to stronger activity. Antioxidant activity is a crucial factor in determining the quality of persimmon wine [13]. This study explores the relationship between phenolic compounds and antioxidant activity in rapeseed flower–persimmon wine through in vitro antioxidant capacity assays combined with computer simulation analyses.

Molecular docking and simulation techniques are pivotal in food chemistry for elucidating compound interactions at molecular resolutions [14]. Molecular docking predicts binding sites and affinities between food components and receptors, providing a foundation for functional ingredient design. Molecular simulations decode the dynamic behavior of these interactions, revealing mechanistic insights into bioactive compounds [15]. These techniques also screen for adverse interactions in food safety evaluations, advancing innovation in food chemistry. Previous studies employed molecular dynamics simulations to investigate the effects of tannin–salivary protein interactions on the volatility of aroma compounds in simulated wine [16]. Additionally, researchers employed isothermal titration calorimetry (ITC) and molecular dynamics simulations to investigate salivary protein–wine flavanol interactions (e.g., catechin, epicatechin, and their mixtures), providing insights into the mechanisms underlying astringency [17]. Furthermore, in vitro antioxidant assays, along with molecular simulations, were conducted to evaluate the antioxidant potential of Eupatorium adenophorum alkaloids and polyphenols [18].

Health care wine has always been a key focus of research, and red bean cedar fruit wine (TCFW) has a long history as a traditional folk health wine, known for its role in boosting immunity and combating aging. Additionally, a new health-oriented persimmon wine has been developed using mixed fermentation of Ganoderma lucidum and persimmon. However, the combination of rapeseed flower and persimmon in wine fermentation has not yet been explored.

This research aims to promote environmentally friendly production methods through natural plant-based fermentation. By studying the mixed fermentation of persimmon and rapeseed flower, the goal was to develop a composite persimmon wine with unique flavor and high nutritional value. The study employed molecular docking, molecular dynamics simulations, and in vitro antioxidant tests to investigate D-glucopyranose–quercetin interactions in persimmon wine. Additionally, volatile flavor compounds in oleoresin-enriched persimmon wine were detected and analyzed using headspace solid-phase microextraction coupled with gas chromatography–mass spectrometry (HS-SPME-GC-MS). The above experiments were conducted to investigate whether mixed fermentation could improve the sensory quality and nutritional composition of persimmon wine and enhance their marketability. The innovative co-fermentation of rapeseed flower and persimmon not only continues the tradition of producing health-oriented wine but also diversifies and expands the range of fermented beverages, achieving a balance between flavor and health benefits and paving the way for modern advancements in traditional fruit wine innovation.

## 2. Materials and Methods

### 2.1. Materials and Instruments

**Figure 1:**
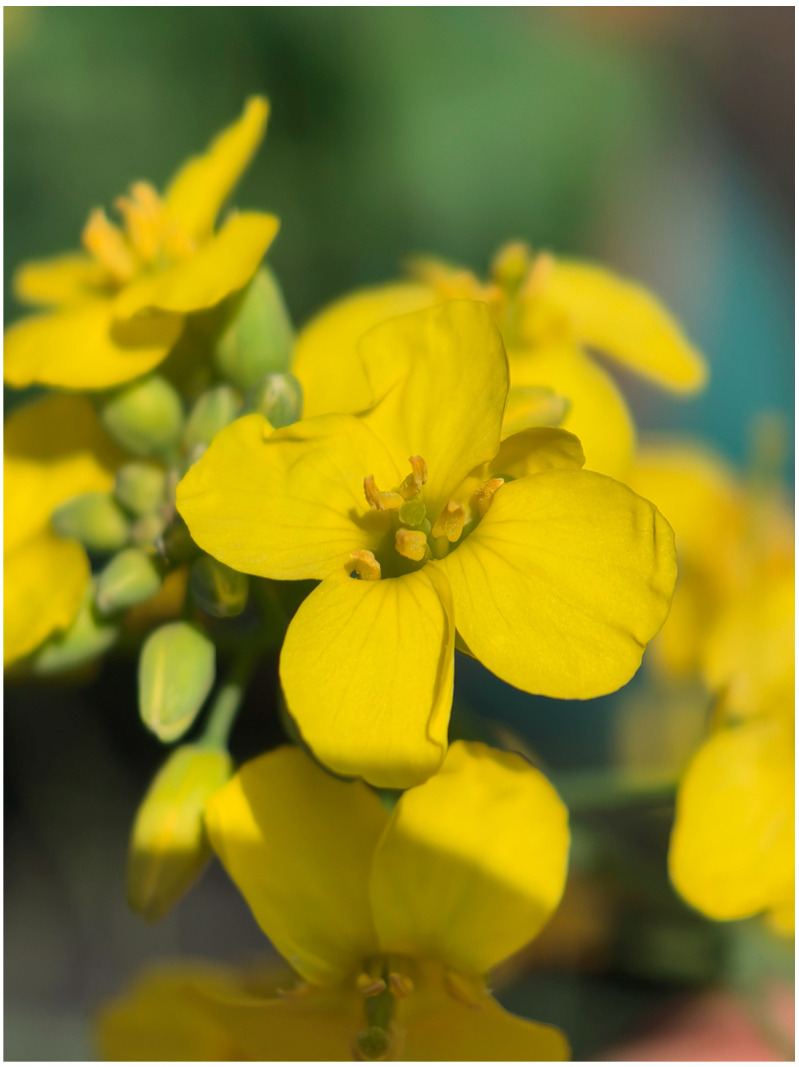
Rape flower in full bloom.

Rapeseed flowers were cultivated and harvested in Dalian, Liaoning Province, as the primary location. Sowing was carried out in mid-March, with consistent irrigation during the germination stage, transitioning to regular watering every five days. The rapeseed flowers were harvested in mid-to-late May. Sampling began during the flowering stage, and fully bloomed, vibrantly colored flowers were selected for sampling, as depicted in Figure 1. The stems were removed, retaining only the anthers and petals for subsequent analysis.

Persimmons, including Luotian sweet persimmons and Luotian improved persimmons, were sourced from Luotian County, Hubei Province, with a soluble solid content (SSC) as high as 25%. Panshan flat persimmons, a specialty of Guanzhuang Town, Jizhou District, Tianjin, had an SSC of approximately 15%. Local persimmons were harvested from persimmon trees on campus in Dalian, Liaoning Province, exhibiting an SSC ranging from 13% to 16%.

The yeast strain used was Angel high-activity dry yeast, obtained from Angel Yeast Co., Ltd., Yichang, Hubei, China. Pectinase was obtained from Weifang, Shandong Sukahan Bio-Engineering Co., Ltd. Sodium nitrite was sourced from Tianjin Damao Chemical Reagent Factory, Tianjin, China. White granulated sugar was sourced from COFCO Sugar Co., Ltd., Chongzuo, China. Hydrochloric acid (HCl) was obtained from Beijing Chemical Works, Beijing, China. Ethanol, potassium chloride (KCl), concentrated sulfuric acid (H_2_SO_4_), aluminum nitrate (Al(NO_3_)_3_), and methanol were procured from Tianjin Kermio Chemical Reagent Co., Ltd., Tianjin, China.

### 2.2. Experimental Methods

#### 2.2.1. Brewing of Rapeseed Flower–Persimmon Fermented Wine

Persimmons and Brassica napus (rapeseed flowers) served as the primary raw materials, with the fermentation process utilizing pomace during brewing [19]. The pomace constitutes 20–30% of the fresh fruit’s total weight. Tannins, pigments, and bioactive compounds (e.g., resveratrol) present in the peel contribute significantly to the product’s distinctive flavor profile. Additionally, the naturally adherent wild yeast flora facilitates spontaneous fermentation, thereby mitigating the environmental impact that would otherwise result from direct pomace disposal.

#### Brewing Process

The rapeseed flower–persimmon wine brewing process comprised the following steps:

Persimmons → cleaning and impurity removal → pulping → the addition of rapeseed flowers → enzymatic hydrolysis (with pectinase) → the addition of SO_2_ → composition adjustment → yeast inoculation → fermentation → racking → aging → clarification → final product.

#### Key Operational Points

Fully ripened persimmons, free from any rot or spoilage, were selected. Freshly harvested rapeseed flowers, retaining intact anthers and petals, were required. We added 0.15‰ pectinase to the persimmon juice and hydrolyzed it at room temperature for 4 h to improve the juice yield. We introduced 0.1 g/L of SO_2_ into the mixture, and then inoculated the mixture with 0.2 g/L of activated Angel yeast, followed by fermentation at (20 ± 1) °C for 7–10 days. Fermentation was terminated when the soluble solid content stabilized and the residual sugar content dropped below 5 g/L. The clarification and aging processes were carried out at 4 °C.

#### 2.2.2. Qualitative Analysis of Monosaccharide Composition of Rapeseed Flower Polysaccharide

Fresh rapeseed flowers (10 g) were oven-dried at 70 °C to a constant weight and then ground into a fine powder using a mortar grinder. Polysaccharide samples (5 mg) were hydrolyzed with 5 mL of 60% (*v*/*v*) trifluoroacetic acid (TFA) solution at 80 °C for 6 h in sealed glass ampoules. The hydrolysate (1 mL) was transferred to a 20 mL glass reactor and evaporated under a nitrogen stream to remove residual TFA. The residue was derivatized with 1.8 mL of anhydrous pyridine and 200 μL of N,O-bis(trimethylsilyl)trifluoroacetamide (BSTFA) containing 1% trimethylchlorosilane (TMCS) at 80 °C for 30 min, and then immediately filtered through a 0.22 μm PTFE membrane prior to GC-MS analysis.

Analysis was conducted using an Agilent 8860-5977B GC-MS from Agilent Technologies Co., Ltd. (Beijing, China) system fitted with an HP-5MS capillary column (30 m × 0.25 mm × 0.25 μm). The injection parameters included a sample volume of 0.1 μL, an injector temperature of 280 °C, a split ratio of 20:1, and a transfer line temperature of 280 °C. The oven temperature program began at 50 °C (held for 2 min), followed by a ramp of 10 °C/min to 150 °C, then 5 °C/min to 250 °C (held for 2 min), and finally, 10 °C/min to 280 °C (held for 2 min). The MS conditions featured an electron ionization (EI) source at 230 °C, an interface temperature of 250 °C, an electron energy of 70 eV, and a mass scan range of *m*/*z* 30–550.

#### 2.2.3. Molecular Docking

Following the qualitative analysis of the monosaccharide composition in rapeseed flower polysaccharides, D-glucopyranose, D-xylopyranose, and D-galactopyranose—the most abundant monosaccharides—were selected. These were combined with three key phenolic compounds from rapeseed flower–persimmon wine, namely quercetin, anthocyanin, and p-coumaric acid. Using PyMOL 3.1, the PDB 3D coordinate files of these six molecules were generated, and their atomic types were validated. Based on the aforementioned molecules, missing hydrogen atoms were added, and the molecular geometries were optimized using the Amber 22. The optimized molecules were then saved in PDB format. The molecular optimization minimized the potential energy, ensuring the molecules adopted their most stable conformations and the optimal binding configurations were determined [20]. Using the optimized molecules, molecular docking experiments were performed between the monosaccharides and phenolic compounds using AutoDock Vina v1.1.2.. Each of the three monosaccharides was individually docked with each of the three phenolic compounds 20 times, and the free binding energy values were recorded for each simulation. Based on the binding energy data from these docking results, D-glucopyranose and quercetin were selected for further in-depth analysis [21].

#### 2.2.4. Molecular Dynamics Simulation

All-atom molecular dynamics (MD) simulations were performed using the AMBER 20 software, with the D-glucopyranose/Quercetin complex as the starting structure. Before running the simulation, the charges of the small molecules were determined using the Hartree-Fock (HF) SCF/6-31G* method in Gaussian 09, facilitated by the Antechamber module. The Generalized Amber Force Field (GAFF) was applied to parameterize the small molecules. Hydrogen atoms were added to each system using the LEaP module, and a truncated octahedral TIP3P water box was placed at a 10 Å distance. Sodium (Na^+^) and chloride (Cl^−^) ions were incorporated to neutralize the system, and the necessary topology and parameter files for the simulation were generated [22].

#### Simulated Conditions

Molecular dynamics (MD) simulations were performed using the AMBER 20 software package. Before initiating the simulations, the system underwent energy minimization, which involved 2500 steps of the steepest descent method, followed by an additional 2500 steps using the conjugate gradient approach. Once minimized, the system was gradually heated from 0 K to 298.15 K under constant volume conditions, with a controlled heating rate applied over a 200 ps period. At 298.15 K, 500 ps of NVT ensemble simulations were conducted to ensure a uniform distribution of solvent molecules within the simulation box. Subsequently, 500 ps of NPT ensemble equilibration simulations were carried out under isothermal–isobaric conditions. Finally, a 50 ns NPT ensemble production simulation was performed for the entire system under periodic boundary conditions. A non-bonded cutoff distance of 10 Å was applied throughout the process. Long-range electrostatic interactions were computed using the particle mesh Ewald (PME) method, while hydrogen bond lengths were constrained via the SHAKE algorithm. Temperature control was achieved using the Langevin thermostat, with a collision frequency (γ) of 2 ps^−1^. The system pressure was maintained at 1 atm, with a time step of 2 fs, and trajectory snapshots were collected every 20 ps for subsequent analysis [23].

#### RMSF Analysis

In molecular dynamics simulations, root mean square fluctuation (RMSF) analysis is typically performed during the post-processing stage. The molecular trajectory output file is created following energy minimization, equilibration, and production simulation steps. The trajectories are then aligned with a reference structure (commonly the initial or mean structure) to eliminate interference from overall molecular movement or rotation during analysis. RMSF values representing the central fluctuations of each atom or residue are calculated using the correlation analysis tool, AMBER. Finally, the RMSF values are mapped to the molecular structure to generate a dynamic heat map.

#### 2.2.5. Determination of Total Antioxidant Capacity (TAC) in Samples Using the FRAP Method

The experiments were carried out according to the total antioxidant capacity (T-AOC) assay kit (Solarbio, Beijing, China). In an acidic environment, the reduction of Fe^3+^-TPTZ (ferric-tripyridyltriazine) to blue-colored Fe^2+^-TPTZ reflects the total antioxidant capacity (TAC) of the substance. A series of standard solutions were prepared, and their absorbance at 593 nm was recorded to generate a standard curve [24]. The reaction mixture, consisting of the persimmon wine sample and distilled water, was thoroughly mixed and incubated at room temperature for 10 min. The absorbance was subsequently measured at 593 nm, and the antioxidant capacity of the sample was quantified as the equivalent standard ion concentration (µmol/mL) producing the same change in absorbance [25].

#### 2.2.6. Determination of Hydroxyl Radical Scavenging Activity in Samples

The experiment followed the protocol provided by the hydroxyl radical scavenging assay kit (Leagene, Beijing, China). The assay is based on the principle that H_2_O_2_/Fe^2+^ generates hydroxyl radicals through the Fenton reaction, which oxidizes Fe^2+^ to Fe^3+^. This reaction results in the conversion of the red-colored o-phenanthroline-Fe^2+^ complex to the colorless o-phenanthroline-Fe^3+^ complex, causing the disappearance of the maximum absorption peak of o-phenanthroline-Fe^2+^ at 536 nm. The absorbance changes in the range of 530–540 nm were measured using a spectrophotometer. From these changes, the variation in the hydroxyl radical content was calculated, and the hydroxyl radical scavenging rate (or activity) of the sample was determined [26].

#### 2.2.7. Determination of DPPH Radical Scavenging Activity in Samples

The experiments were based on the DPPH free radical scavenging assay kit (Leagene, Beijing, China). The assay operates on the principle that the DPPH radical is a stable, nitrogen-containing radical with an unpaired electron. When dissolved in ethanol, DPPH forms a purple-colored solution with a strong absorption peak at 517 nm. When another substance donates an electron to pair with the unpaired electron, the solution undergoes decolorization. The extent of decolorization is directly proportional to the number of electrons accepted, and the reduction in absorbance indicates the sample’s capacity to scavenge nitrogen radicals [27]. The absorbance changes at 517 nm were measured using a spectrophotometer to determine the variation in DPPH radical content. The DPPH radical scavenging rate or scavenging activity of the sample was then calculated accordingly [28].

#### 2.2.8. Determination of Vitamin C Content in Samples

The experiment was based on the vitamin C free radical scavenging assay kit (Leagene, Beijing, China). The detection principle involves the reaction of ammonium molybdate with reduced vitamin C under strongly acidic conditions and the presence of phosphate ions, generating a blue-colored compound [29]. Within a defined concentration range, absorbance values exhibit a linear relationship with vitamin C concentration. The absorbance was measured at 760 nm using a spectrophotometer to quantify the vitamin C content [30].

The experimental materials consisted of distilled water, centrifuge tubes, a visible spectrophotometer, a thermostatic water bath, a low-temperature centrifuge, and 1 mL glass cuvettes.

#### 2.2.9. Determination of Flavoring Substances in Persimmon Wine

The volatile flavor compounds in rapeseed flower–persimmon wine were quantitatively analyzed using headspace solid-phase microextraction coupled with gas chromatography–mass spectrometry (HS-SPME-GC-MS). The experimental procedure is described as follows [31]:

1. Microextraction conditions:

The SPME fiber (SUPELCO) was 50/30 μm PDMS/DVB/CAR, Stableflex. The fiber was preconditioned at a temperature of 250 °C for 300 s. The insertion depth was adjusted to 20 mm, and the fiber coating extended by 12 mm. Samples were placed in 20 mL headspace vials and preheated to 40 °C. The extraction was performed at 60 °C over 2400 s, with an insertion depth of 15 mm and a coating extension length of 12 mm. Stirring was carried out at 300 r/min for 600 s. The desorption temperature was maintained at 270 °C for 300 s, with an insertion depth of 20 mm and a coating extension length of 12 mm.

2. Gas chromatography conditions:

A capillary column Agilent 19091s-433 HP-5MS (30 m × 0.25 mm × 0.25 μm) was used, from Agilent Technologies Co., Ltd., Beijing, China. The temperature profile followed a programmed sequence: initially held at 40 °C for 5 min, increased at 2 °C/min to 70 °C, held for 2 min at 70 °C, ramped at 3 °C/min to 120 °C, at 5 °C/min to 150 °C, and finally, at 10 °C/min to 230 °C, held for 2 min. The flow rate was 1.4 mL/min. High-purity helium (99.999%) was used as the carrier gas at a column flow rate of 1.4 mL/min. A split injection mode was employed, with a split ratio of 5:1, and the septum purge flow was maintained at 3 mL/min. The inlet temperature was 270 °C.

3. Mass spectrometry conditions:

The ionization source was an EI source with an electron energy of 70 eV. The ion source temperature was maintained at 230 °C, the quadrupole temperature at 150 °C, and the transfer line temperature at 280 °C. Data acquisition was performed in Scan/SIM mode, with a scan mass range of 35–550. A solvent delay of 1 min was applied [32].

4. Qualitative and quantitative analysis of aroma components

For qualitative analysis, the HS-SPME-GC-MS coupling technique was used for the identification and characterization of aroma components. The retrieved profiles were matched against the NIST2002 standard mass spectral library using the instrumental setup of the Xcalibur workstation and further compared with relevant references for confirmation.

For quantification, only aroma compounds with a similarity index of over 80% were considered valid. The relative content of each aroma compound was calculated semi-quantitatively using the internal standard method, with 2-octanol serving as the internal standard.

5. Evaluation and Analysis of Fragrance Composition

The contribution of each aroma compound to the overall flavor of the samples was evaluated based on the relative odor activity value (ROAV), with larger ROAVs indicating a greater contribution to the overall flavor of the samples. The ROAVs were classified as follows: 0 < ROAV < 0.1, potential flavor compounds; 0.1 < ROAV < 1, flavor-modifying compounds; 1 < ROAV < 100, key flavor compounds. The ROAV of each compound was calculated using Equation (1).

(1) $$ROAV=\frac{OAV_{1}}{OAV_{max}}\times 100=\frac{C_{1}}{T_{1}}\times \frac{C_{max}}{T_{max}}\times 100$$

where OAV_1_, C_1_, and T_1_ represent the odor activity value, mass concentration (mg/L), and sensory threshold (mg/L) for each aroma compound; OAV_max_, C_max_, and T_max_ represent the odor activity value, mass concentration (mg/L), and sensory threshold (mg/L) of the aroma compound with the highest contribution to the overall flavor, respectively.

## 3. Results

### 3.1. Qualitative Analysis of Monosaccharide Composition in Rapeseed Flower Polysaccharides

Polysaccharides from rape flower were hydrolyzed using trifluoroacetic acid and subsequently derivatized by silylation. Gas chromatography–mass spectrometry (GC-MS) was employed to characterize the monosaccharide components. The total ion chromatogram revealed 34 peaks, including solvent peaks and derivatized components. Further details are presented in Figure S1. Through NIST database searches, the rapeseed flower polysaccharides were determined to contain *α*- and *β*-pyran rings of glucose isomers. The four primary silylated monosaccharide derivatives identified were glucose, galactose, arabinose, and xylose. The main silylated derivatives of rapeseed flower polysaccharides are summarized in Table 1.

### 3.2. Molecular Docking Results

**Figure 2:**
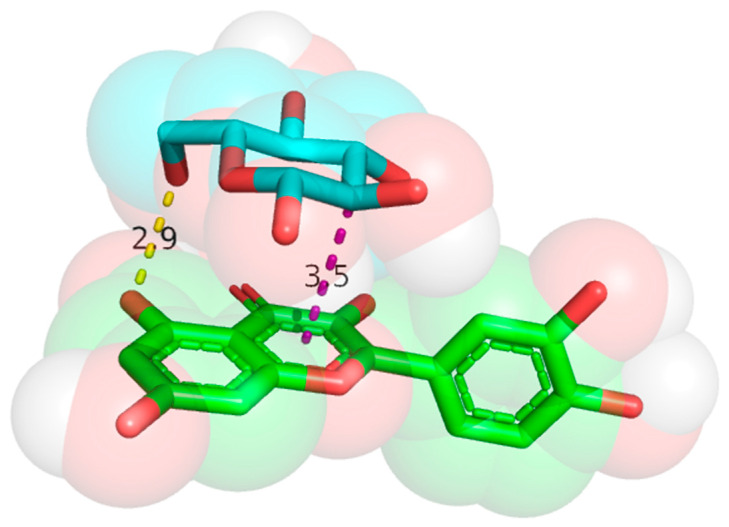
D-glucopyranose/quercetin molecular docking diagram.

Three monosaccharides were selected from Table 1, in descending order of content, based on their relative abundances. D-glucopyranose, D-xylopyranose, and D-galactopyranose were selected for molecular docking. These monosaccharides were docked 20 times each with three key phenolic compounds in rapeseed flower–persimmon wine: quercetin, anthocyanin, and p-coumaric acid. Virtual screening was performed based on the binding free energy rankings. The binding free energy values obtained from the docking simulations are presented in Table S1. Using AutoDock Vina v1.1.2, molecular docking between quercetin and D-glucopyranose yielded binding free energy values of −4.7 and −4.8 kcal/mol. The docking results were visualized and analyzed using PyMOL 3.1, as shown in Figure 2. When D-glucopyranose interacts with quercetin, hydrogen bonds are formed between the hydroxyl group of D-glucopyranose and the phenolic hydroxyl group of quercetin, with bond distances of 2.9 Å and 3.5 Å, respectively.

The docking results reveal multiple hydrogen bonds and hydrophobic interactions between D-glucopyranose and quercetin, demonstrating a strong binding affinity.

### 3.3. Molecular Simulation Dynamics Analysis

The molecular simulation results show that the D-glucopyranose/quercetin complex remained stable throughout the simulation, exhibiting relatively low interaction energy, which supports potential synergistic effects during fermentation. The root mean square deviation (RMSD) obtained from the molecular dynamics simulations reflects the movement of the complex. Higher and more fluctuating RMSD values indicate intense motion, while lower and more stable RMSD values suggest smoother motion.

**Figure 3:**
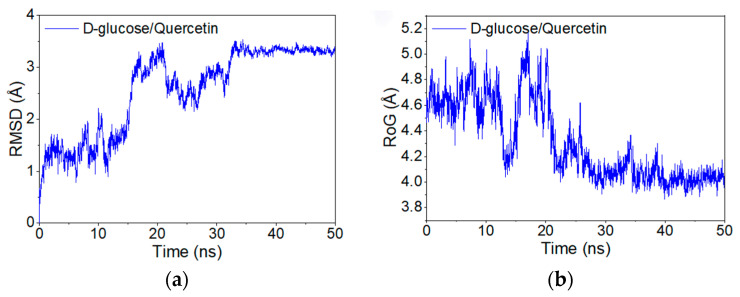
(**a**) Root mean square deviation (RMSD) of D-glucopyranose/quercetin complex during molecular dynamics simulation. (**b**) Variation in the radius of gyration of the composite with time.

As shown in Figure 3a, the RMSD of the D-glucopyranose/quercetin complex was monitored over a 50 ns molecular dynamics simulation. Significant fluctuations in the RMSD were observed during the first 35 ns, suggesting continuous conformational adjustments. After 35 ns, the RMSD stabilized at approximately 3.5 Å, indicating that the complex achieved conformational convergence and exhibited stable binding and motion in the later stages of the simulation.

Additionally, the radius of gyration (Rg) of the complex was calculated during the simulation. As shown in Figure 3b, the Rg was relatively large at the beginning but gradually decreased as the simulation progressed. This reduction in Rg reflects an increase in system compactness, suggesting that the binding between D-glucopyranose and quercetin became increasingly favorable.

**Figure 4:**
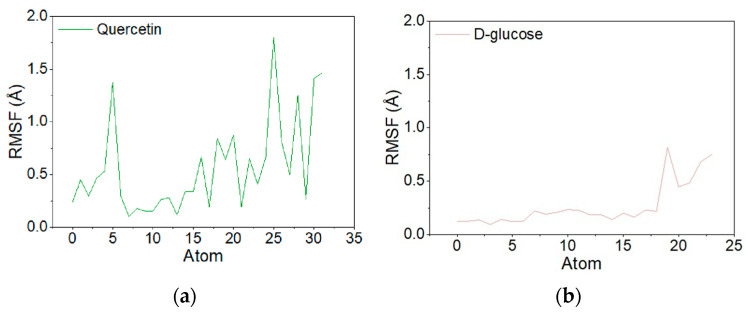
(**a**) Quercetin RMSF analysis. (**b**) D-glucopyranose RMSF analysis.

Figure 4 indicates that the fluctuation of atoms in the quercetin molecule, when combined with D-glucopyranose, was significantly higher, with RMSF values ranging from 0.2 Å to 1.8 Å. This highlights notable differences in the dynamic stability of various atoms within the quercetin molecule, suggesting greater molecular flexibility and dynamic behavior. In contrast, the RMSF values for the D-glucopyranose molecule were relatively low, fluctuating between 0.1 Å and 0.6 Å, indicating reduced atomic mobility and higher stability. Overall, the quercetin molecule demonstrated greater flexibility during the simulation, while D-glucopyranose appeared more rigid and stable.

**Figure 5:**
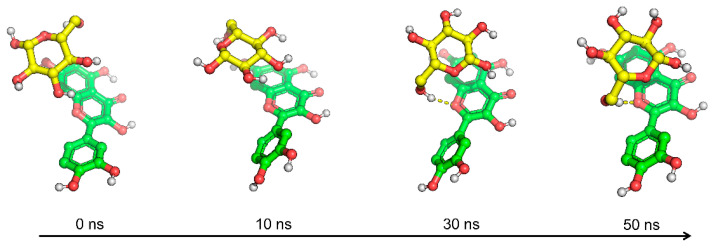
Conformation diagrams of D-glucopyranose and quercetin at 0 ns, 10 ns, 30 ns, and 50 ns.

The conformational states of the D-glucopyranose/quercetin complex at 0 ns, 10 ns, 30 ns, and 50 ns are illustrated in Figure 5. Throughout the simulation, D-glucopyranose adheres to quercetin, with the attachment becoming progressively closer over time. At 30 ns and 50 ns, the hydroxymethyl group of D-glucopyranose forms hydrogen bonds with quercetin, indicating strong binding between the two molecules.

### 3.4. Analysis of IRI Hydrogen Bonding Force

**Figure 6:**
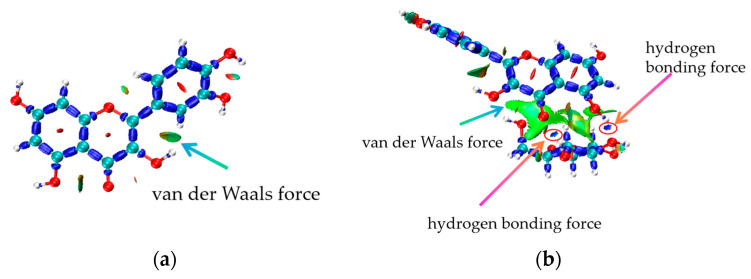
(**a**) Analysis of hydrogen bond forces of quercetin. (**b**) Analysis of hydrogen bond forces after D-glucopyranose and quercetin binding.

As shown in Figure 6a,b, hydrogen bonds primarily form between the hydroxyl groups of D-glucopyranose and the phenolic hydroxyl groups of quercetin after binding. These hydrogen bonds stabilize the quercetin molecule and its binding complex. Once stabilized, the phenolic hydroxyl groups of quercetin are more likely to release hydrogen atoms, enhancing its antioxidant activity. Additionally, electronic interactions occur between the hydroxyl groups of D-glucopyranose and the phenolic hydroxyl groups of quercetin following hydrogen bond formation, altering the electronic environment of the quercetin molecule.

### 3.5. Analysis of RDG Hydrogen Bonding Force

**Figure 7:**
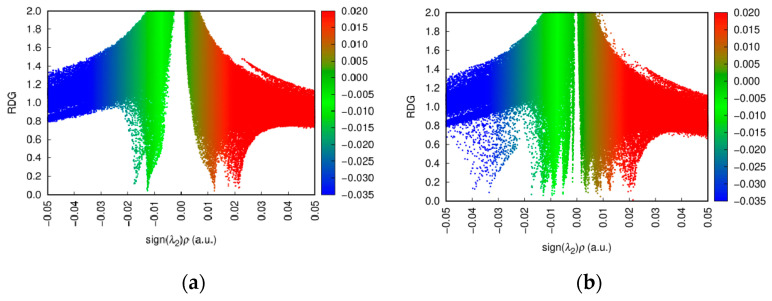
(**a**) RDG analysis of quercetin. (**b**) RDG analysis of D-glucopyranose and quercetin complex. Blue region indicates strong attractive interactions, typically hydrogen bonding or electronegativity driven attraction. Green region indicates weak van der Waals interactions (weak attraction in neutral regions). Red region indicates strong repulsive interactions, typically hard or electrostatic repulsion between nuclei.

By analyzing the RDG (reduced density gradient) before and after the binding of quercetin with D-glucopyranose, it can be observed that the attractive interactions shown in Figure 7b are enhanced compared to those in Figure 7a. Specifically, the following observations can be made:

1. The distribution in the region of negative sign(λ_2_)ρ becomes denser, with a slightly extended range.
2. More blue regions emerge in the low-RDG areas, representing specific strong interaction zones within the attractive region. This indicates a significant increase in strong non-covalent attractive interactions, predominantly hydrogen bonding.

Overall, both the intensity and range of the non-covalent attractive interactions are visibly enhanced.

### 3.6. Electron Density Distribution (VMD Imaging)

**Figure 8:**
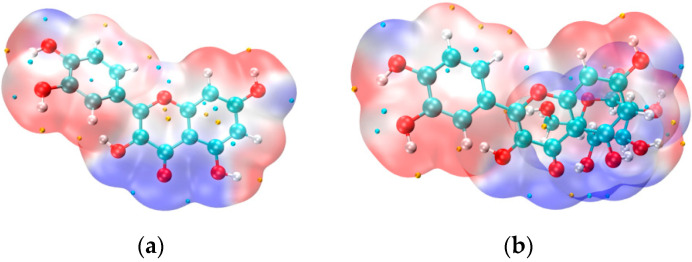
(**a**) Quercetin VMD imaging. (**b**) VMD imaging of D-glucopyranose and quercetin complex.

Figure 8a shows the electron density distribution of quercetin, while Figure 8b illustrates a more extended electron cloud with pronounced blue regions (representing negative charge) that transition more smoothly into the surrounding areas. This indicates a broader electron density distribution and changes in the overall charge distribution after binding.

From the color differences, it is evident that the blue regions in Figure 8b (quercetin after binding) are reduced compared to Figure 8a. This suggests a decrease in the electronegativity of quercetin, particularly around its phenolic hydroxyl groups. The reduced electronegativity reflects a lower electron cloud density in these regions, making the hydrogen atoms more likely to dissociate and scavenge free radicals.

The increased tendency for hydrogen atom dissociation enhances quercetin’s free radical scavenging ability, thereby improving its antioxidant activity. Additionally, the changes in electronegativity after binding may adjust the electronic environment of quercetin, stabilizing intermediate products and further increasing its antioxidant capacity.

### 3.7. In Vitro Antioxidant Assay

The antioxidant activity of rapeseed flower–persimmon wine is a critical factor determining its overall quality. To evaluate the impact of rapeseed flower addition on the quality of persimmon wine, four in vitro antioxidant activity assays were performed on the rapeseed flower–persimmon wine.

**Figure 9:**
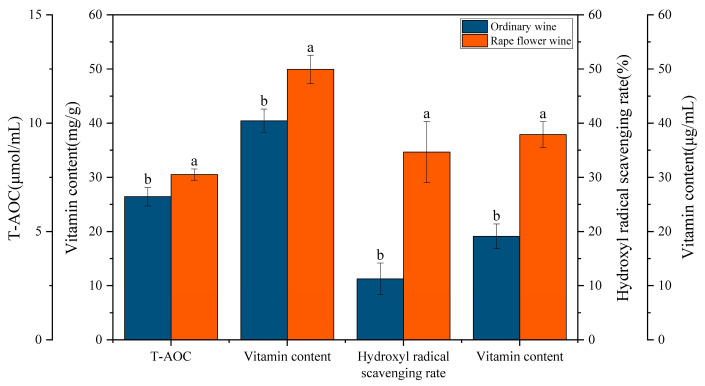
Antioxidant performance of ordinary persimmon wine and rape flowers persimmon wine. Error lines indicate standard errors of three biological replicates; different lower case letters indicate significant differences, *p* < 0.01.

The results from four in vitro antioxidant activity assays demonstrate that the rapeseed flower-enriched Luotian sweet persimmon wine exhibited a 1.98-fold increase in DPPH free radical scavenging activity and a 2.06-fold increase in hydroxyl radical scavenging activity compared to regular persimmon wine (Figure 9). Additionally, its vitamin C content and total antioxidant capacity increased by 1.37-fold and 1.16-fold, respectively.

These findings underscore the significant antioxidant advantages of rapeseed flower–persimmon wine, highlighting its superior health value compared to regular persimmon wine. The co-fermentation of rapeseed flower with persimmon significantly enhanced the wine’s antioxidant potential, aligning well with molecular simulation results. This consistency between experimental and computational findings provides theoretical support and a strong research basis for the development of functional fermented wines.

### 3.8. Composition of Flavoring Substances in Persimmon Wine

Headspace solid-phase microextraction coupled with gas chromatography–mass spectrometry (HS-SPME-GC-MS) was employed to identify and evaluate aroma compounds in the two persimmon wines, as summarized in Table 2. A total of 19 volatile compounds were identified in pure persimmon wine and yellow rape persimmon wine, consisting of 6 alcohols, 6 esters, 1 acid, and 6 other compounds.

The aroma descriptions and sensory thresholds of these flavor compounds were classified based on the literature. As shown in the table, the levels of some alcohols with rich aroma characteristics increased significantly. For instance, butane-2,3-diol, which has a molasses-like aroma, showed a 6.39-fold increase. Additionally, hexan-1-ol, which has fruity and floral notes, and 3-Methylacetic acid-butan-1-ol were absent in pure persimmon wine but were present in the yellow rape–persimmon wine, with their contents increasing significantly. Esters contribute pleasant floral and fruity aromas, playing an essential role in shaping the aromatic profile of wine. According to the table, the content of ethyl hexanoate, which imparts sweet, fruity, and cellar-like aromas, increased by 6.24 times, while the content of ethyl octanoate with fruit and brandy aroma and ethyl decanoate with coconut aroma also increased by 2.22 times and 9.41 times, respectively. The concentrations of certain hydrocarbons with distinct odors, as well as (NE)-N-[1-(2-methoxy-phe-nyl)propan-2-ylidene]hydroxylamine, which has earthy and burnt sensory attributes, decreased significantly in persimmon wine after the addition of yellow rapeseed flower, with (NE)-N-[1-(2-methoxyphenyl)propan-2-ylidene]hydroxylamine content reduced by 14.7 times. The interaction between yellow rapeseed flower and the flavor substances produced during persimmon fermentation resulted in a reduction of undesirable flavor compounds, creating a softer and smoother flavor profile in the persimmon wine.

The numbers and contributions of key, modified, and potential flavor compounds differed between the two types of persimmon wine (Table 3). There were two key flavor compounds (1 ≤ ROAV < 100) in the pure persimmon wine and four in the canola–persimmon wine, which served as the main contributors to the overall aroma of persimmon wine. Modified flavor compounds (0.1 < ROAV < 1) were identified in two categories in the pure persimmon wine and only one in the canola–persimmon wine. There were two potential flavor compounds (0 < ROAV < 0.1) included in the pure persimmon wine and four in the canola–persimmon wine. The key flavor compounds with the highest ROAVs in the pure persimmon wine and canola–persimmon wine were ethyl octanoate and ethyl hexanoate, respectively. Phenethyl alcohol was present in both persimmon wines but differed in concentration. This indicates that persimmon wines, with the addition of canola, contain flavor compounds unique to each type, allowing them to retain their distinctive flavor profiles.

## 4. Conclusions

This study comprehensively investigated the interaction mechanism between D-glucopyranose and quercetin in Olea europaea-enriched persimmon wine and its potential effects on wine quality through a multifaceted approach, including molecular docking, molecular dynamics simulations, and in vitro antioxidant experiments. Additionally, the volatile flavor compounds in two types of persimmon wine (pure persimmon wine and oleaginous persimmon wine) were qualitatively and quantitatively analyzed using headspace solid-phase microextraction (HS-SPME) coupled with gas chromatography–mass spectrometry (GC-MS). The results show that D-glucopyranose formed a stable complex with quercetin through hydrogen bonding and hydrophobic interactions. Notably, hydrogen bonding enhanced the molecular stability of quercetin while reducing the bond energy of its phenolic hydroxyl groups, thereby facilitating hydrogen atom release for antioxidant activity and significantly improving the overall antioxidant capacity.

In vitro experiments further demonstrated that rape flower-enriched persimmon wine exhibited superior antioxidant properties compared to pure persimmon wine. Specifically, its DPPH radical scavenging activity and hydroxyl radical scavenging activity increased 1.98-fold and 2.06-fold, respectively, while its vitamin C content and total antioxidant capacity increased 1.37-fold and 1.16-fold, respectively. These results indicate that the addition of Olea europaea not only enhanced the functional composition of persimmon wine and optimized its antioxidant properties but also improved its flavor and storage stability. HS-SPME-GC-MS analysis revealed that esters, alcohols, and aldehydes were the primary components of persimmon wine aroma, and persimmon wines with varying levels of canola addition exhibited marked differences in key compound content, subsequently influencing aroma complexity and flavor balance. Overall, these findings provide robust data and a theoretical foundation for understanding the role of rapeseed flower in modulating persimmon wine aroma.

## Figures and Tables

**Table 1:** GC-MS analysis of silylated main derivatives of rapeseed flower polysaccharide.

Number	Peak Time/min	Molecular Weight (AMU)	Monosaccharide IUPAC Name	Monosaccharide Abbreviation	Relative Content%
1	17.888	438.23	(3R,4S,5S,6R)-6-(hydroxymethyl)oxane-2,3,4,5-tetrol	D-glucopyranose	5.26%
2	18.14	554.24	(3R,4S,5S,6R)-6-(hydroxymethyl)oxane-2,3,4,5-tetrol	D-glucopyranose	1.53%
3	18.36	378.171	(3R,4S,5R)-oxane-2,3,4,5-tetrol	D-xylopyranose	34.00%
4	19.04	438.211	(3R,4S,5S)-oxane-2,3,4,5-tetrol	L-arabinopyranose	1.02%
5	19.188	452.22	(3R,4S,5R,6R)-6-(hydroxymethyl)oxane-2,3,4,5-tetrol	D-galactopyranose	5.40%
6	19.57	496.253	(3R,4S,5R,6R)-6-(hydroxymethyl)oxane-2,3,4,5-tetrol	D-galactopyranose	5.45%
7	20.275	466.206	(3R,4S,5S,6R)-6-(hydroxymethyl)oxane-2,3,4,5-tetrol	D-glucopyranose	40.40%
8	21.207	554.24	(2S,3S,4S,5R)-3,4,5,6-tetrahydroxyoxane-2-carboxylic acid	D-glucopyranuronic acid	2.05%
9	21.504	540.26	(3R,4S,5S,6R)-6-(hydroxymethyl)oxane-2,3,4,5-tetrol	D-glucopyranose	4.88%

**Table 2:** Flavoring substances in two persimmon wines and their flavor characteristics.

Serial Number	Name of Substance	Amount Present (μg/L)		ROAV		Flavor Threshold (μg/L)	Fragrance Description
		Pure Persimmon Wine	Rapeseed Flower– Persimmon Wine	Pure Persimmon Wine	Rapeseed Flower– Persimmon Wine		
1	butane-2,3-diol	32.34	206.78	0.0000072	0.000046	4,500,000	Molasses
2	hexan-1-ol	—	107.8	—	0.0135	8000	Fruity
3	phenethyl alcohol	3089.94	1546.44	0.221	0.11	14,000	Saussurea costus
4	heptan-1-ol	—	24.598	—	—	—	Grassy flavor
5	tetradecan-1-ol	—	33.32	—	—	—	Molasses
6	3-Methylacetic acid-butan-1-ol	—	808.5	—	0.027	30,000	Fruity
7	Ethyl acetate	—	45.08	—	0.003	17,000	Fruity
8	Ethyl hexanoate	178.365	1117.3	2.347	14.701	76	Fruity
9	Ethyl benzoate	—	12.152	—	0.008	1434	Cherry scent
10	Diethyl succinate	—	80.36	—	0.0008	100,000	Fruity
11	Ethyl octanoate	746.79	1661.1	3.112	6.921	240	Brandy flavor
12	Ethyl decanoate	36.26	341.09	0.363	3.411	100	Coconut scent
13	octanoic acid	549.78	485.12	0.037	0.0323	15,000	Fruity
14	2,2,4,4,6,6-hexamethyl-1,3,5,2,4,6-trioxatrisilinane	267.54	225.45	—	—	—	Pungent odor
15	2,2,4,4,6,6,8,8,10,10-decamethyl-1,3,5,7,9,2,4,6,8,10-pentaoxapentasilecane	245.98	158.76	—	—	—	Pungent odor
16	bis[[dimethyl(trimethylsilyloxy)silyl]oxy]-dimethylsilane	255.78	115.64	2.56	1.16	100	Pungent odor
17	Nonanal	47.04	78.41	—	—	—	Citrus zest
18	Decanal	37.24	28.42	0.372	0.284	100	Citrus zest
19	(NE)-N-[1-(2-methoxyphenyl)propan-2-ylidene]hydroxylamine	545.86	37.142	—	—	—	Earthy, flavor

Aroma thresholds are from the literature; ‘—’ indicates that no relevant information was found.

**Table 3:** ROAVs of characteristic flavor compounds in wine.

Name of Substance	ROAV	
	Pure Persimmon Wine	Rapeseed Flower–Persimmon Wine
butane-2,3-diol	0.0000072	0.000046
hexan-1-ol	—	0.0135
phenethyl alcohol	0.221	0.11
3-Methylacetic acid-butan-1-ol	—	0.027
Ethyl hexanoate	2.347	14.701
Ethyl octanoate	3.112	6.921
Ethyl decanoate	0.363	3.411
octanoic acid	0.037	0.0323
bis[[dimethyl(trimethylsilyloxy)silyl]oxy]-dimethylsilane	2.560	1.160

Aroma thresholds are from the literature; ‘—’ indicates that no relevant information was found.

## Data Availability

The original contributions presented in this study are included in the article/Supplementary Materials. Further inquiries can be directed to the corresponding author.

## Supplementary Materials

The following supporting information can be downloaded at https://www.mdpi.com/article/10.3390/foods14101804/s1, Figure S1. Total Ion Flow Chromatogram. Table S1. Molecular docking free binding energy value.
