# Supplementary material for: Analysis of Antioxidant Activity and Volatile Components in Rapeseed Flower-Enriched Persimmon Wine
Source: Foods. 2025 May 19;14(10):1804. doi: 10.3390/foods14101804 (PMC12111799; doi:10.3390/foods14101804)
Supplement: Supplementary file 1 [file foods-14-01804-s001.zip › foods-3613402-supplementary.pdf]

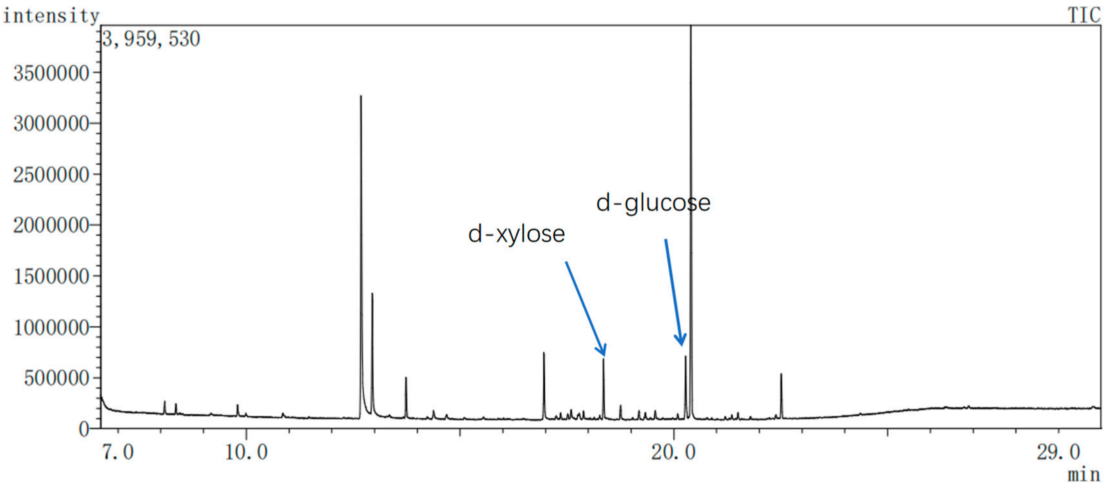

Figure A1 Total Ion Flow Chromatogram

Table S1. molecular docking free binding energy value

Macromolecule	Ligand	Binding energy（kcal/moL）
	Anthocyanin	-1.6
D-Galactopyranose	Quercetin	-1.4
	p-Coumaric acid	-1.4
	Anthocyanin	-1.6
D-Glucopyranose	Quercetin	-2.1
	p-Coumaric acid	-1.4
	Anthocyanin	-1.3
D-xylopyranose	Quercetin	-1.7
	p-Coumaric acid	-1.2
